# Epithelioid angiosarcoma arising in schwannoma of the kidney: report of the first case and review of the literature

**DOI:** 10.1186/s12957-016-0789-5

**Published:** 2016-02-03

**Authors:** G. Iannaci, M. Crispino, P. Cifarelli, M. Montella, I. Panarese, A. Ronchi, R. Russo, G. Tremiterra, R. Luise, P. Sapere

**Affiliations:** 1 Division of Pathology, S. Maria del Popolo degli Incurabili Hospital ASL Na1, Naples, Italy; 2 Division of Urology, S. Maria del Popolo degli Incurabili Hospital ASL Na1, Naples, Italy; 3 Division of Pathology, School of Medicine, University Federico II, Naples, Italy

**Keywords:** Kidney angiosarcoma, Epithelioid angiosarcoma, Kidney schwannoma, Angiosarcoma arising in schwannoma, Literature review

## Abstract

**Background:**

Schwannoma and angiosarcoma are infrequent pathologies that have been rarely reported in the kidney. Angiosarcoma is an uncommon malignant tumor presenting a recognizable vascular differentiation. It can develop in any site but the most common locations include the skin, soft tissues, breast, bone, liver, and spleen while renal localization has been very rarely reported in the literature. Schwannoma is a benign peripheral nerve sheath tumor composed of cells with the immunophenotype and ultrastructural features of differentiated Schwann cells. It has a wide anatomical distribution but the most frequent locations include subcutaneous tissues of the extremities and the head and neck region and the retroperitoneal and mediastinal soft tissues. The occurrence of an angiosarcoma in a pre-existing schwannoma is an extremely rare event with <20 cases reported in worldwide literature. In the present study, a renal case of angiosarcoma arising in schwannoma is presented with a detailed review of the pertinent literature.

**Case Presentation:**

A 56-year-old man was admitted with a few days history of lower back pain and hematuria. Abdominal ultrasound showed a mass inside the left renal medulla. Subsequent imaging investigations with computed tomography and magnetic resonance confirmed the presence of the lesion and showed a pulmonary metastasis.

**Conclusions:**

The final histopathological examination led to the diagnosis of epithelioid angiosarcoma arising in a schwannoma. The patient came to death a few months later due to a massive hemothorax. To the best of our knowledge, the present is the first case of an angiosarcoma arising in a schwannoma of the kidney.

## Background

Schwannoma (also known as neurilemmoma) is a benign peripheral nerve sheath tumor composed of cells with the immunophenotype and ultrastructural features of differentiated Schwann cells. It occurs in patients of any age with a slight predilection for adults [1]. The anatomic distribution is wide but the most frequent locations include subcutaneous tissues of the extremities and the head and neck region and the retroperitoneal and mediastinal soft tissues [1]. In most cases, it presents itself as a sporadic solitary lesion, but some cases are associated with the hereditary syndrome neurofibromatosis type 2 [2]. The etiology of schwannoma seems to be linked to loss of expression of the protein merlin that performs a number of critical functions such as contact-dependent inhibition of proliferation, cellular adhesion, and transmembrane signaling [3]. Diagnosis may be suspected on the basis of the clinical features of the lesion and the possible relationship with a nerve but it always requires pathological investigation. The gross appearance is that of a nodular, well-circumscribed, and encapsulated mass with a pink to yellow cut surface. Histologically, the tumor is composed of spindle cells with indistinct cell borders and moderately abundant eosinophilic cytoplasm. The most characteristic histologic feature is the nuclear palisading and the presence of eosinophilic masses circumscribed by rows of nuclei formerly known as Verocay bodies. There are two tissue types: Antoni A (hypercellular) and Antoni B (hypocellular with relatively abundant loose tissue). Many distinct variants of schwannoma have been described: ancient, plexiform, cellular, melanotic, microcystic, and epithelioid. By immunohistochemistry, tumor cells express S100, vimentin, calretinin, basal lamina components, and calcineurin. Schwannoma very rarely recurs after complete surgical excision, that is almost always curative, and malignant transformation is extremely rare [1]. All cases of malignant transformation reported in literature have occurred in sporadic schwannoma, and the great majority of cases consisted of a malignant peripheral nerve sheath tumor [4]. No case has been reported in patients with neurofibromatosis. The most common symptoms observed in patients with malignant change in schwannoma included pain or rapid enlargement of a pre-existing lesion. These symptoms are rare in schwannoma and should therefore support the suspicion of a malignant transformation. Sarcomas generally do not arise in peripheral nerve sheath tumors, with the exception of angiosarcoma [5]. The majority of malignant peripheral nerve sheath tumors and all the cases of angiosarcoma arising in a schwannoma have an epithelioid morphology [6]. Up to date, there is no explanation for this finding.

Angiosarcoma is an uncommon malignant tumor presenting a recognizable vascular differentiation. It accounts for only 2 to 4 % of soft tissue sarcomas [7] and occurs mainly in the adulthood and elderly, but occasional cases in children have been reported [8]. It can develop in any site but the most common locations include the skin, soft tissues, breast, bone, liver, and spleen, while the rare cases seen in children occur especially in mediastinum including the heart and pericardium. Known risk factors include previous radiation therapy and traumas, but the etiology of this neoplasm remains unknown. Recent studies have shown the role of genes involved in the receptor protein tyrosine kinase pathway, in particular the upregulation of MYC, KIT, and RET and downregulation of CDKN2C in post-radiation angiosarcomas [9]. Clinically, the symptomatology depends substantially on the location of the lesion and is related to the effect of the mass that can compress adjacent anatomical structures, to the anemia resulting from blood loss and to lymphedema; other symptoms often observed include pain, weight loss, and asthenia. The gross appearance is characterized by extensive hemorrhagic areas and infiltrating margins. Epithelioid morphology is rare in cutaneous angiosarcomas while it is frequently seen in those arising in soft tissues and visceral locations. Regardless of the histological features, angiosarcoma is considered high grade by definition [10]; the prognosis is very poor; soft tissue forms present more than 50 % of mortality within 1 year of diagnosis [11] because of the strong tendency for recurrence and the almost constant occurrence of disseminated metastases. At older ages, larger tumor size and retroperitoneal location are poor prognostic factors.

We describe a case of a complex renal lesion that consists of two components changing abruptly within the tumor: a larger, malignant neoplasm diagnosed as an epithelioid angiosarcoma and a minor benign neural tumor diagnosed as schwannoma. It is well known that angiosarcomas can develop in neurofibromas and malignant peripheral nerve sheath tumors, especially in patients with von Recklinghausen’s disease. The occurrence of an angiosarcoma in a schwannoma is a very rare event as only 14 cases have been reported in the literature. To our knowledge, the present is the first case of an angiosarcoma arising in a schwannoma of the kidney.

## Case presentation

**Fig. 1:**
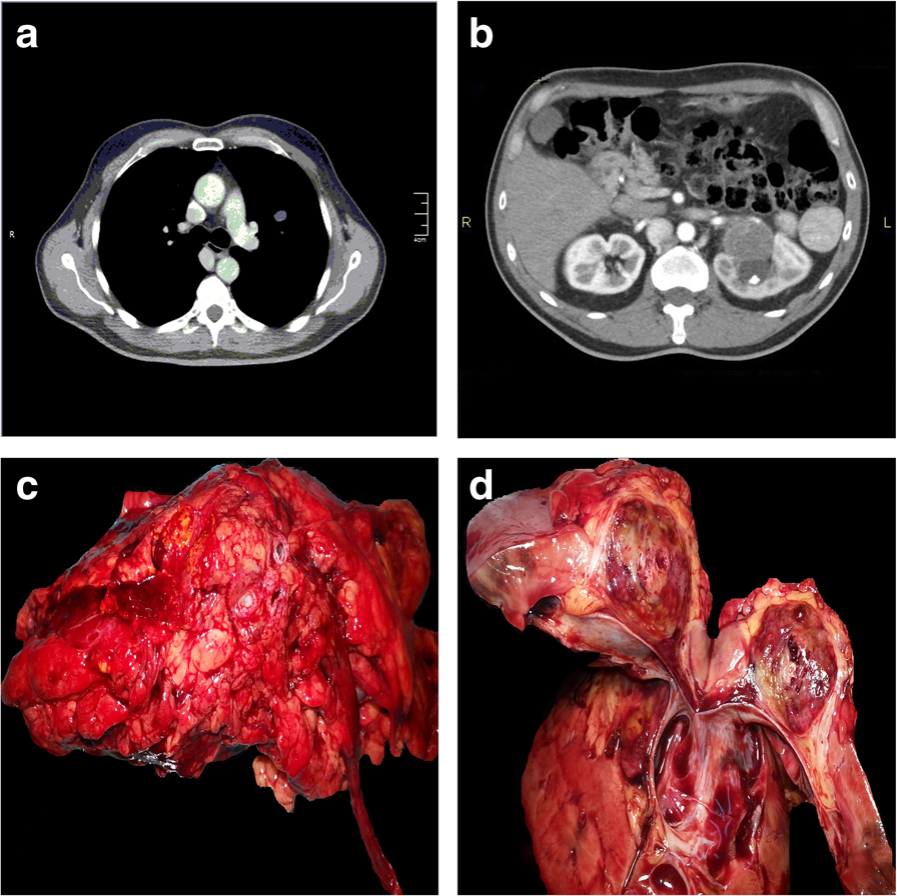
**a** Computed tomography: imaging identifies an intraparenchymal nodule in the left lung. **b** Computed tomography: imaging identifies the presence of a nodular mesonephric hypodense lesion characterized by post contrastographic enhancement. **c** Gross imaging: the kidney shows an irregular profile with granulating appearance of the hilum. (**d**) Gross imaging: sectioned tumor appears hemorrhagic, soft, and reddish

A 56-year-old man with a history of non-insulin dependent diabetes mellitus was referred to the surgical department because of the onset of lower back pain and hematuria that occurred the previous day. The patient was not a smoker and denied alcohol consumption; his family history was unremarkable and there was no other complaint. The ultrasound of the left kidney revealed the presence of stones in the renal pelvis causing obstruction of the ureteral meatus and consequent dilatation of the upper calix. During the examination, the presence of a solid neoplasm was noticed that involved the renal medulla reaching the hilum area without signs of hilar infiltration. For further investigations, the patient underwent CT (computed tomography) and MR (magnetic resonance) that showed the slight enhancement and extensive necrotic areas of the lesion and the existence of a solid nodule with same features in the left lung, likely due to metastases (Figs. 1a-1b). A lombotomic total nephrectomy was performed, and the patient had an uneventful post-operative recovery.

**Fig. 2:**
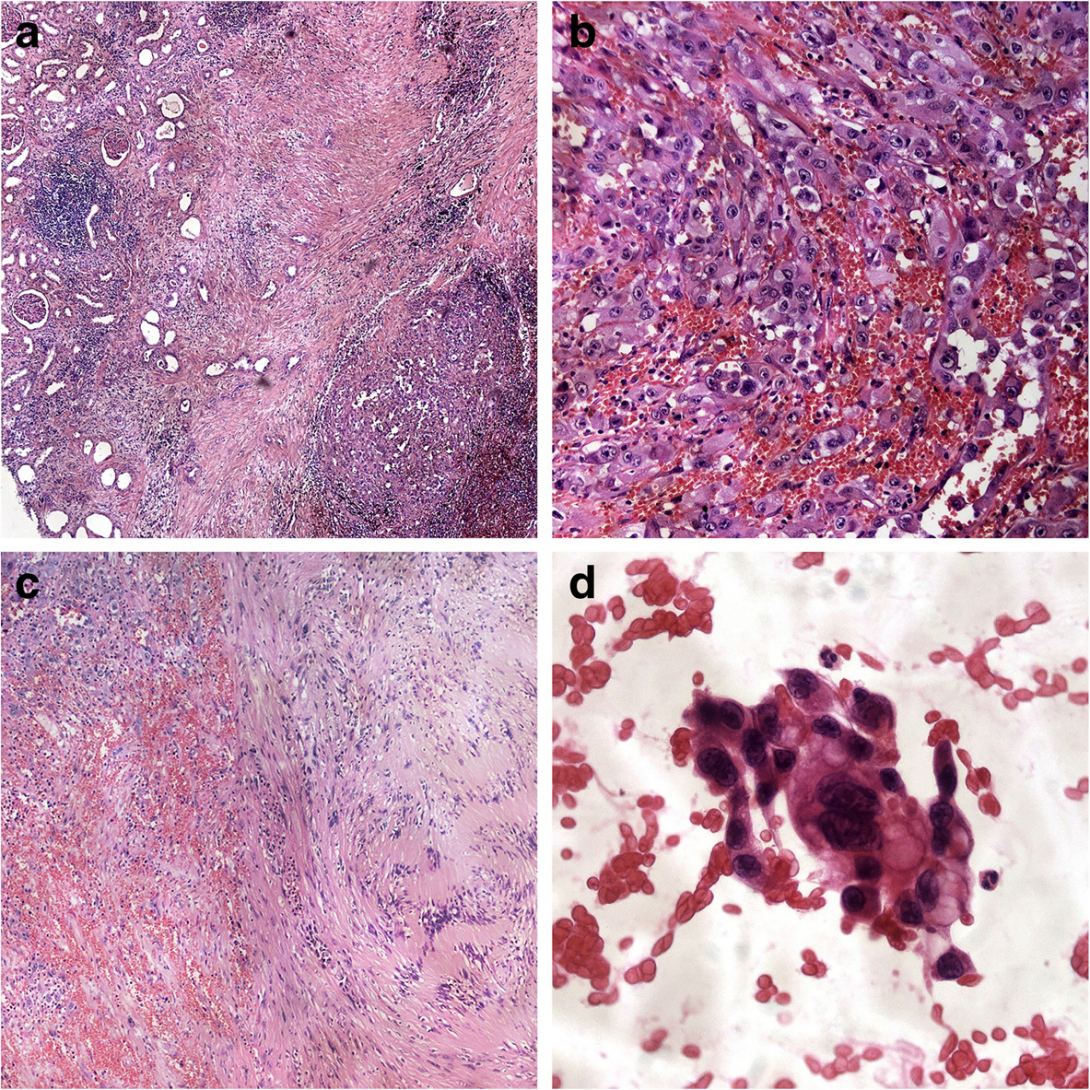
**a** H&E staining (×10): numerous neoplastic vascular channels (*left*) that infiltrates the renal parenchyma (*right*). **b** H&E staining (×20): neoplastic cells forming vascular structures with epithelioid features and highly atypical nuclei. **c** H&E staining (×10): malignant cells of angiosarcoma (*left*) juxtaposed to benign spindle cells population of schwannoma (*right*). **d** H&E staining (×40): fine needle aspiration biopsy of the pulmonary lesion shows neoplastic cells

**Fig. 3:**
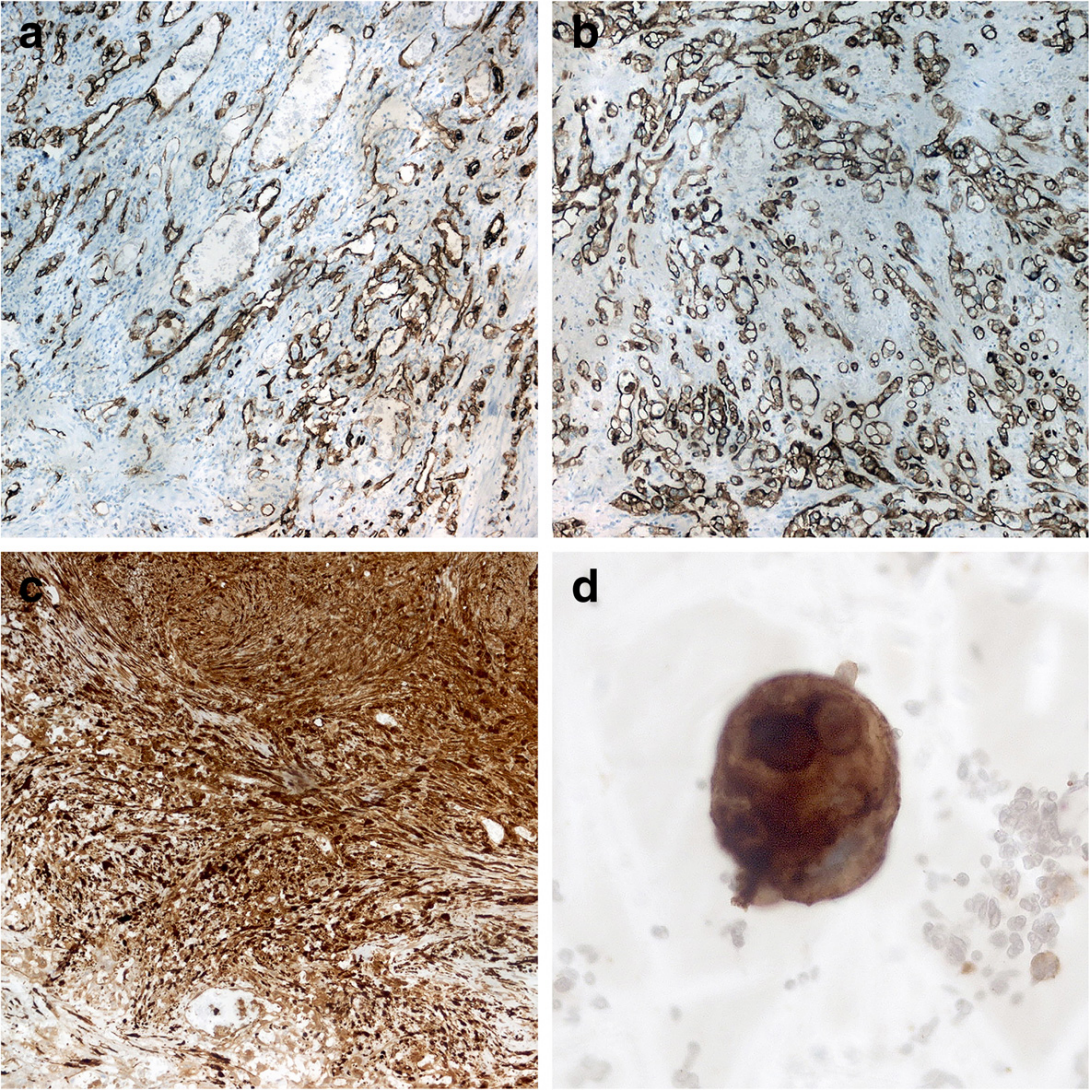
**a**, **b** Immunohistochemical staining (×10): neoplastic cells show immunopositivity for CD34 and cytokeratin. **c** Immunohistochemical staining (×10): schwannoma cells with strong, diffuse S100 immunoreactivity. **d** Immunohistochemical staining (×40): neoplastic cells present in fine-needle aspiration biopsy of the pulmonary lesion are CD34 positive

Grossly, the kidney presented irregular profile due to a hilar lesion measuring 4 cm × 2.5 cm. On cut sections, it was highly hemorrhagic, soft, and reddish with infiltrating borders (Figs. 1c-1d). The tumor was extensively sampled. Microscopic examination was performed on paraffin-embedded sections stained with hematoxylin and eosin (Figs. 2 and 3). Histopathological examination showed a proliferation of canalicular structures of various sizes, sometimes with some degree of cystic changes, lined by atypical cells with epithelioid features, deeply invading the renal parenchyma and the perinephric fat. Many of these structures contained red blood cells giving the idea that it was a vascular lesion. In some fields was observed an almost solid growth pattern with erythrocytes trapped in thin spaces between neoplastic cells. Marked cellular pleomorphism, enlarged and hyperchromic nuclei, irregular nuclear contour, prominent nucleoli, and frequent mitotic figures were also evident. In the context of this lesion, few fields showed a quite different morphology consisting of spindle cells arranged in palisading fashion without cellular atypia or invasive features. An immunohistochemical study was performed on formalin-fixed paraffin-embedded tissue block to define the histogenesis of the lesion. Pre-diluted antibodies produced by Ventana-Roche were used, directed against pan-cytokeratin (clone AE1/AE3/PCK26; mouse monoclonal), CD34 (clone QBEnd/10; mouse monoclonal), factor VIII (rabbit polyclonal), Ki67 (clone 30-9; mouse monoclonal), S100 (clone 4C4.9; mouse monoclonal), EMA (clone E29; mouse monoclonal), and CD10 (clone SP67; rabbit monoclonal). The malignant population was reactive for pan-cytokeratin, CD34, factor VIII, partially positive for Ki67 (positivity in about 40 % of neoplastic cells) and negative for S100, EMA and CD10, therefore was consistent with epithelioid angiosarcoma. Otherwise, the benign spindle cells population was positive for S100 and negative for Ki67 and epithelial and endothelial markers. It was so interpreted as residuals of pre-existent schwannoma. In view of these morphological and immunohistochemical findings, a diagnosis of primary renal epithelioid angiosarcoma probably arising in schwannoma was made. A CT-guided fine-needle aspiration cytology of the pulmonary lesion was subsequently performed that showed CD31-positive atypical epithelioid cells in the context of numerous erythrocytes, confirming the diagnosis of lung metastasis of angiosarcoma. During the post-operative period, the patient has not been subjected to antineoplastic therapy because of poor general health and he came to exitus a few months after diagnosis, because of a massive hemothorax caused by lung metastasis.

## Conclusions

Angiosarcoma is an infrequent neoplasm with a very poor prognosis which is why it has been very rarely described in the kidney. The diagnosis of this lesion is extremely difficult if not impossible when considering just the clinical and radiological features. So, it is often referred to post-operative time, and pathological examination remains the gold standard. The occurrence of an angiosarcoma in a pre-existent schwannoma is an exceptionally rare event with an unknown pathogenesis. In view of the rarity of the lesion, an extensive review of the literature was undertaken through a MEDLINE search using the search terms “primary renal schwannoma/primary schwannoma of the kidney,” “primary renal angiosarcoma/primary angiosarcoma of the kidney,” and “angiosarcoma arising in schwannoma.” Only reports in English have been taken into account. In Tables 1, 2, and 3 are listed the cases reported in the literature specifying the source and the clinical features of each case.

**Table 1:** Cases of primary schwannoma of the kidney

Author	Sex	Age (years)	Side	Location	Year
Verze et al. [12]	Male	59	Right	Parenchymal	2014
Wang et al. [13]	Male	66	Left	Parenchymal	2013
Mikkilineni et al. [14]	Female	36	Right	Parenchymal	2013
Yang et al. [15]	Female	40	Left	Hilum	2012
Nayyar et al. [16]	Female	44	Right	Hilum	2011
Sfoungaristos et al. [17]	Female	55	Left	Parenchymal	2011
Qiguang et al. [18]	Male	34	Right	Hilum	2010
Gobbo et al. [19]	Female	27	Left	Hilum	2008
Gobbo et al. [19]	Female	35	Right	Hilum	2008
Gobbo et al. [19]	Female	39	Left	Parenchymal	2008
Hung et al. [20]	Female	36	Left	Parenchymal	2008
Alvarado-Cabrero et al. [21]	Male	45	Left	Parenchymal	2000
Alvarado-Cabrero et al. [21]	Female	40	Left	Parenchymal	2000
Alvarado-Cabrero et al. [21]	Male	84	Right	Parenchymal	2000
Alvarado-Cabrero et al. [21]	Female	14	Right	Parenchymal	2000
Singer et al. [22]	Female	70	Left	Capsule	1996
Bezzi et al. [23]	Male	60	Right	Hilum	1996
Kitagawa et al. [24]	Male	51	Left	Hilum	1990
Ma et al. [25]	Male	67	Right	Parenchymal	1990
Somers et al. [26]	Female	55	Left	Parenchymal	1988
Phillips et al. [27]	Male	56	Left	Hilum	1955

**Table 2:** Cases of primary angiosarcoma of the kidney

Author	Sex	Age (years)	Side	Year	Clinical presentation
Zhang et al. [28]	Male	52	Left	2014	Leg pain and flank pain
Qayyum et al. [29]	Female	86	Right	2014	Fatigue, dizziness, weight loss
Liu et al. [30]	Male	75	Right	2014	Gross hematuria
Sabharwal et al. [31]	Male	67	Left	2013	Flank pain, weight loss
Chaabouni et al. [32]	Male	59	Right	2013	Flank pain, gross hematuria
Singh et al. [33]	Male	83	Left	2012	Acute chest pain, dyspnea
Douard et al. [34]	Male	60	Right	2012	Hodgkin’s lymphoma history
Zenico et al. [35]	Male	56	Left	2011	Hodgkin’s lymphoma history
Papadimitriou [36]	Male	68	Left	2009	Flank pain
Fukunaga [37]	Male	61	Left	2009	Hypertension
Leggio et al. [38]	Male	60	Left	2006	After trauma
Akkad et al. [39]	Male	58	Right	2006	Asymptomatic
Johnson et al. [40]	Male	50	Left	2002	Flank pain, hemoptysis
Aksoy et al. [41]	Male	55	Left	2002	Spontaneous rupture
Aydogdu et al. [42]	Male	77	Left	1999	Gross hematuria, flank pain
Cerilli et al. [43]	Male	67	Right	1998	Gross hematuria, flank pain
Tsuda et al. [44]	Male	77	Left	1997	Gross hematuria, renal failure
Mordkin et al. [45]	Male	75	Left	1997	Weight loss, fever
Hiratsuka et al. [46]	Female	59	Right	1997	Hematuria
Martinez-Piñeiro et al. [47]	Male	66	Left	1995	Asthenia
Kern et al. [48]	Male	69	Left	1995	Flank pain, hematuria, weight loss
Kern et al. [48]	Male	56	Left	1995	Hematuria
Adjiman et al. [49]	Male	36	Right	1990	Flank pain, cough, hemoptysis
Desai et al. [50]	Male	54	Left	1989	Flank pain, microhematuria
Cason et al. [51]	Male	46	Left	1987	Flank pain, weight loss, fever
Terris et al. [52]	Male	47	Left	1986	Flank pain
Allred et al. [53]	Male	67	Right	1981	Flank pain, hematuria
Askari et al. [54]	Male	24	Right	1980	Hematuria
Peters et al. [55]	Male	74	Left	1974	Weight loss

**Table 3:** Cases of angiosarcoma arising in schwannoma

Author	Sex	Age (years)	Location	Year
Mahajan et al. [56]	Male	41	Neck, vagus nerve	2014
Li et al. [57]	Male	67	Right abdominal adrenergic nerve	2012
Li et al. [57]	Male	38	Right inguinal sciatic nerve	2012
Li et al. [57]	Male	55	Left neck, vagus nerve	2012
Lee et al. [58]	Male	73	Left thigh, sciatic nerve	2007
Ito et al. [59]	Male	66	Intracranial vestibular nerve	2007
McMenamin et al. [60]	Female	74	Right neck, vagus nerve	2001
McMenamin et al. [60]	Female	40	Right thigh, sciatic nerve	2001
McMenamin et al. [60]	Female	17	Right neck, phrenic nerve	2001
McMenamin et al. [60]	Female	39	Right buttock	2001
Ruckert et al. [61]	Male	50	Right neck, vagus nerve	2000
Mentzel et al.	Female	73	Right neck, vagus nerve	1999
Mentzel et al.	Male	63	Right neck, vagus nerve	1999
Trassard et al. [62]	Male	65	Right thigh, sciatic nerve	1996

Twenty-one cases of renal schwannoma have been reported in literature (Table 1) [12–27]. Tumors involved patients ranging in age from 14 to 84 years, with a median age of 48 years and a slight predominance in females (male to female ratio of 0.75:1). Renal schwannomas were mainly located in the parenchyma and less frequently in the hilum.

Twenty-nine cases of primary renal angiosarcoma have been reported in literature [28–55]. The median age of patients was 61.5 years with an age range comprised from 24 to 86 years. The great majority of tumors have been found in males with a male to female ratio of 13.5:1 (27 males and 2 females). Angiosarcoma was seen to arise preferably in the left kidney (right to left ratio of 0.5:1). The most common symptoms reported were the classical symptoms due to a renal mass like flank pain and hematuria, while more rarely there were symptoms related to the presence of metastasis at time of diagnosis like cough, hemoptysis, and dizziness. Three cases were asymptomatic, and the lesions have been found as incidental findings during diagnostic tests conducted for other reasons.

Angiosarcomas arise very rarely in the context of a pre-existing schwannoma. To the best of our knowledge, only 14 cases have been reported in literature to date [56–62]. In all these cases, an angiosarcomatous component had an epithelial morphology. Patients were aged between 17 and 74 with a median age of 55 and a male to female ratio of 1.8:1. The locations of the lesions included the neck, leg, buttock, intracranial, abdominal cavity, and inguinal region; no case has been previously reported in the kidney.

### Consent

Written informed consent was obtained from the patient for publication of this case report and any accompanying images. A copy of the written consent is available for review by the Editor-in-Chief of this journal.
